# Improved Kalman Filter Method for Measurement Noise Reduction in Multi Sensor RFID Systems

**DOI:** 10.3390/s111110266

**Published:** 2011-10-28

**Authors:** Ki Hwan Eom, Seung Joon Lee, Yeo Sun Kyung, Chang Won Lee, Min Chul Kim, Kyung Kwon Jung

**Affiliations:** Department of Electronic Engineering, Dongguk University, 26, Pil-dong 3-ga, Jung-gu, 100-715, Seoul, Korea; E-Mails: acousticjoon@naver.com (S.J.L.); moravito@nate.com (Y.S.K.); whitenight85@naver.com (C.W.L.); kmch0405@naver.com (M.C.K.); kwon@dgu.ac.kr (K.K.J.)

**Keywords:** smart RFID tags, Kalman filter, neural network, multi-sensing environment, measurement noise reduction

## Abstract

Recently, the range of available Radio Frequency Identification (RFID) tags has been widened to include smart RFID tags which can monitor their varying surroundings. One of the most important factors for better performance of smart RFID system is accurate measurement from various sensors. In the multi-sensing environment, some noisy signals are obtained because of the changing surroundings. We propose in this paper an improved Kalman filter method to reduce noise and obtain correct data. Performance of Kalman filter is determined by a measurement and system noise covariance which are usually called the R and Q variables in the Kalman filter algorithm. Choosing a correct R and Q variable is one of the most important design factors for better performance of the Kalman filter. For this reason, we proposed an improved Kalman filter to advance an ability of noise reduction of the Kalman filter. The measurement noise covariance was only considered because the system architecture is simple and can be adjusted by the neural network. With this method, more accurate data can be obtained with smart RFID tags. In a simulation the proposed improved Kalman filter has 40.1%, 60.4% and 87.5% less Mean Squared Error (MSE) than the conventional Kalman filter method for a temperature sensor, humidity sensor and oxygen sensor, respectively. The performance of the proposed method was also verified with some experiments.

## 1. Introduction

In the field of Radio Frequency Identification (RFID) technology, a tremendous variety of novel RFID sensor tags has emerged. The RFID sensor tags also known as smart RFID tags are able to measure and compute data from the environmental such as temperature, humidity, oxygen concentration, pressure, tampering, shock, *etc.* These three functions of intelligent RFID tags: sensing, computation, and communication, can be combined into a single and small device. The great needs for new sensing solutions is further highlighted by the fact that legislation, regulatory and quality demands are setting requirements for certain branches such as pharmaceuticals, explosives, transportation of dangerous goods, foods, *etc.* Cold chain compliance is a key requirement for pharmaceuticals, hospital transfusions, clinical trials, foods and perishable items. RFID can be used to fight counterfeiting and can provide the electrical pedigree of various products [1]. As modern RFID systems become incredibly complex and demand for smart RFIDs is increasing, and RFID tag monitoring systems based on a single sensor are often unable to meet the new societal needs. Smart RFID tags combined with multi sensors and attached on a box or package of diverse products can provide integrated information to managers and customers by combining various sensor data from its sensing materials. For example, integrated data from temperature, humidity and oxygen sensors from an RFID tag attached on food or vegetable packaging can provide the conditions of products and can protect people from consuming spoiled food. Until now, the smart RFID tags with single sensors provided this integrated information by using several tags. However, if various sensors are integrated into a single RFID tag, low-cost, -power and -area can be realized to implement the RFID system [2].

Multi-sensors such as resistive, capacitive and inductive type sensors can be combined into smart RFID tags. These diverse sensor data can represent the freshness and vitality of living organism. In a multi-sensing RFID tag environment, the tags can obtain data of multi-sensors using both just one port or several ports. In a multi-sensor system that correlates noises caused by multiple sensors, accuracy of sensor data is one of the most important factors to evaluate the monitoring system. The Kalman filter has been widely applied to solve the noise problem of measurement systems [3]. The Kalman filter can optimally estimate the states of the model with known parameters. However, variance of the measurement noise which should be evaluated from empirical noise data is hard to derive in most actual measurement environments. If the statistics of the measurement noise are unknown, the Kalman filter cannot guarantee optimality and freedom from divergence problems [4]. In order to solve this problem, we propose an adaptive filtering method of colored noise based on the Kalman filter employing neural network. The neural network of this method can evaluate the states of the plant even without actual measurement noise statistics. Computed statistical noise variables are used as an input of the neural network. Certain noise covariance of each sensor from a lot of measured data is applied to the target value. The improved method presented in this paper is applied to reduce measurement noise of empirical sensor data from a multi-sensing environment. Various simulation and experimental results demonstrate the possibility and good performance of the proposed method. This paper is organized as follows: in Section 2, a multi-sensing environment which causes disturbances and sensor data noises is described. Section 3 represents common the Kalman filter method and the proposed method using a neural network. Section 4 shows several simulation results to compare the performance of common the Kalman filter method and the proposed method. Section 5 gives the experimental results of the improved Kalman filter on an experimental system using a TMS320F28X EVM to evaluate the performance. Some concluding remarks are given in Section 6.

## 2. Multi-Sensing Environment

**Figure 1.**
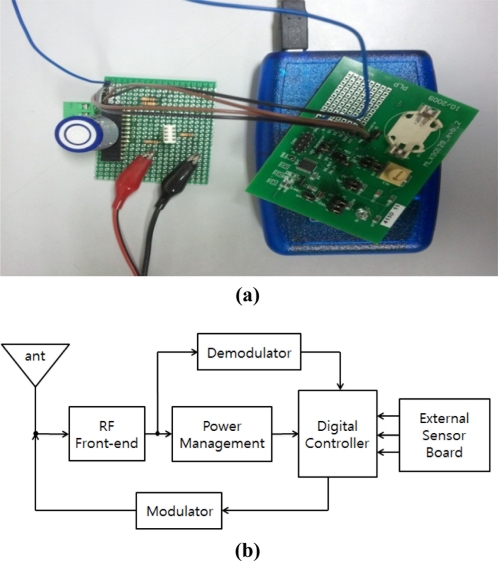
Measurement system configuration based on EVB 90129. **(a)** Picture. **(b)** Block diagram.

**Figure 2.**
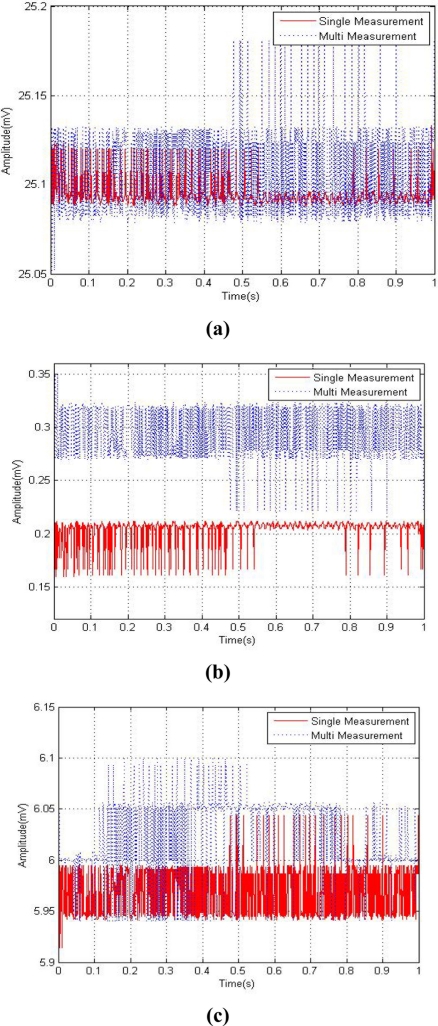
Compare sensor data between the single (solid line) and multi-sensing environment (dotted line). **(a)** Temperature sensor. **(b)** Humidity sensor. **(c)** Oxygen sensor.

When a system designer organizes a multi sensor system, each sensor may not work properly because of some factors that disturb their work. Those factors are noise and interference, which are caused by the measurement system in a multi-sensing environment. The multi-sensing RFID system used was composed of a EVB90129 by Melexis Microeletronic Systems and a sensor board combined with a temperature sensor, humidity sensor and oxygen sensor as shown in Figure 1. The EVB 90129 is an RFID sensing platform and it was read by a RFID reader connected with a PC through a USB port to measure sensor data and plot figures. Figure 2 shows graphs of the empirical data obtained from a single sensor and each sensor in a multi-sensing environment. Each data set from the single sensor and the multi-sensing environment was measured under the same condition in the same surroundings.

Figure 2(a–c) show measurement data obtained from the temperature sensor, humidity sensor and oxygen sensor, respectively. The solid line of Figure 2 represents single measurement data, and the dotted line shows the measurement data in the multi-sensing environment.

The measurement data of the multi-sensing environment has more noise and disturbances than the single measurement as shown in Figure 2. The multi-sensing environment measurement system cannot obtain accurate data from each sensor because several sensors are connected to a single device. Also, the oxygen sensor presents more sensor data measurement noise than the other two sensors data [see Figure 2(c)]. Therefore, a novel noise reduction technique was required for a smart RFID tag multi-sensing environment to obtain accurate data and information. The measurement noise variance of each sensor was calculated from the above data to use as a target value for the neural network.

## 3. Improved Kalman Filter

The Kalman filter requires that all the plant dynamics and noise processes be known exactly and when the noise processes are zero it means there is white noise. If the theoretical behavior of a filter and its actual behavior are not matched, a divergence problem will occur. When the error covariance is computed from the actual error in the measurement, satisfactory results are obtained without divergence. Noise covariance in the Kalman filter acts as in the role of controlling the bandwidth and modulates the Kalman gain. Abnormal choice of noise covariance is one of the most important factors which make Kalman filters diverge. The purpose of the proposed method is the estimation of the noise covariance by using neural networks to prevent divergence of Kalman filter.

### 3.1. The Kalman Filter

The Kalman Filter is an algorithm which makes optimal use of imprecise data in a linear system with noises to continuously update the best estimate of the system’s current state. Kalman filter theory is based on a state-space approach in which a state equation models the dynamics of the signal generation process and an observation equation models the noisy and distorted observation signal.

The random variables *w_k_* and *υ_k_* represent the process and measurement noise respectively. They are assumed to be independent of each other, white, and with normal probability distributions [5,6]:

(1) $$x_{k+1}={\mathrm{Ax}}_{k}+{\mathrm{Bu}}_{k}+w_{k}$$

(2) $$z_{k}={\mathrm{Hx}}_{k}+\upsilon_{k}$$

The matrix *A* in the difference Equation (1) relates the state at the previous time step *k*−1 to the state at the current step *k*, in the absence of either a driving function or process noise. The matrix *B* relates the optional control input. The matrix *H* in the measurement Equation (2) relates the state to the measurement *z_k_*:

(3) P(w)∼N(0,Q)

(4) P(υ)∼N(0,R)

In practice, Q represents the process noise covariance and R is the measurement noise covariance. In deriving the Kalman filter formulation, we begin with the goal of finding an equation that computes an a posteriori state estimate as a linear combination of an *a priori* estimate and a weighted difference between an actual measurement and a measurement prediction [5,7].

The time update equations are responsible for projecting forward (in time) the current state and error covariance estimates to obtain the *a priori* estimates for the next time step. The measurement update equations are responsible for the feedback, *i.e.*, for incorporating a new measurement into the *a priori* estimate to obtain an improved a posteriori estimate:

(5) $${\hat{x}}_{k/k}={\hat{x}}_{k/k-1}+K_{k}\left\{z_{k}-{H\hat{x}}_{k/k-1}\right\}$$

We define *x̂*_*k*/*k*−1_ to be our *a priori* estimate at step k from the previous prediction of *x_k_*, and *x̂*_*k*/*k*_ to be our *a posteriori* state estimate at step k given measurement *x_k_*. *z_k_* is the noisy observation vector. *Hx̂*_*k*/*k*−1_ is a presumption of the pre-measurement value. *x̂*_*k*/*k*_ can be expressed as in Equation (6):

(6) $${\hat{x}}_{k/k-1}={A\hat{x}}_{k-1/k-1}+{\mathrm{Bu}}_{k}$$

The Kalman gain, *K_k_*, is selected to minimize the *a posteriori* state estimate by incorporating the measurement. *E*{*e_k/k_e_k/k_^T^*} is the equation for the Kalman filter, the goal of this equation is finding a Kalman gain, *K_k_*. E is an operator of expectation value. Equation (7) is the equation that expresses the *a posteriori* estimate errors:

(7) $$e_{k/k}=x_{k}-{\hat{x}}_{k/k}$$

Derivation of the equation of estimate error covariance to compute the Kalman gain, *K_k_*, is presented by Equation (8):

(8) $$E\left\{e_{k/k}\;e_{k/k}^{T}\right\}=P_{k/k-1}+K_{k}{\mathrm{HP}}_{k/k-1}H^{T}K_{k}^{T}+K_{k}{\mathrm{RK}}_{k}^{T}-P_{k/k-1}H^{T}K_{k}^{T}-K_{k}\;{\mathrm{HP}}_{k/k-1}$$

The above expected value is equivalent to minimizing the trace of the *a posteriori* estimate covariance matrix. The trace is minimized when the matrix derivative is zero, as in Equation (9). Solving Equation (9) for *K_k_* yields the Kalman gain as shown in Equation (10). This gain is the optimal Kalman gain *K_k_*:

(9) $$\frac{\partial E\left\{e_{k/k}\;e_{k/k}\;e_{k/k}^{T}\right\}}{\partial K_{k}}=2{\mathrm{HP}}_{k/k-1}H^{T}K_{k}^{T}+2{\mathrm{RK}}_{k}^{T}-{\mathrm{HP}}_{k/k-1}-{\left\{P_{k/k-1}H^{T}\right\}}^{T}=0$$

(10) $$K_{k}=P_{k/k-1}H^{T}{\left\{{\mathrm{HP}}_{k/k-1}H^{T}+R\right\}}^{-1}$$

The *a posteriori* estimate covariance matrix *P_k/k_* is presented in Equation (11):

(11) $$P_{k/k}=E\left\{e_{k/k}\;e_{k/k}^{T}\right\}=\left(I-K_{k}H\right)P_{k/k-1}$$

The *a priori* estimate covariance matrix of the next step from the Equation (12) step is shown as:

(12) $$P_{k+1/k-1}=E\left\{e_{k+1/k-1}\;e_{k+1/k-1}^{T}\right\}={\mathrm{AP}}_{k/k}A^{T}+Q$$

**Figure 3.**
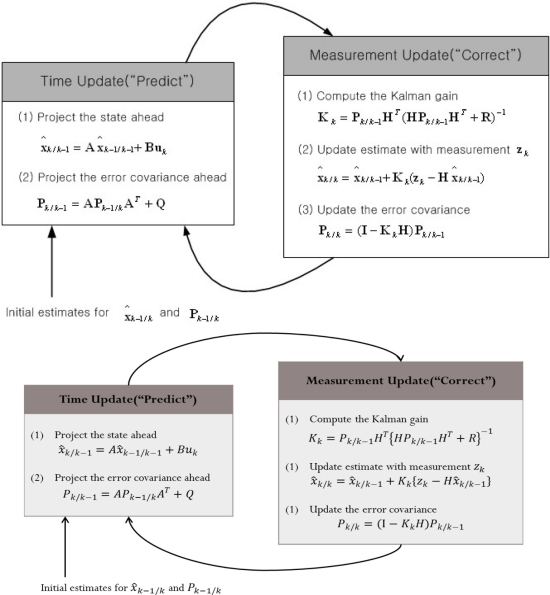
The operation of the Kalman filter.

The complete operation of the Kalman filter is described in Figure 3 [8]. The Kalman filter estimates a process by using a form of feedback control, in other words the filter estimates the process state at same time and then obtains feedback. In common parlance, the equations for the Kalman filter can be divided into two groups: time update equations and measurement update equations. The time update equations can also be thought of as predictor equations, while the measurement update equations can be thought of as corrector equations [9]. Indeed the final estimation algorithm resembles that of a predictor-correct or algorithm for solving numerical problems as shown in Figure 3.

### 3.2. Improved Kalman Filter Using a Neural Network

The measurement equation from the system state equation is shown in Equation (13):

(13) $$z_{k}={\mathrm{Hx}}_{k}+v_{k}$$

When we develop the Equation (13) in the measurement noise *υ_k_* aspects, the Equation results in Equation (14):

(14) $$\upsilon_{k}=z_{k}-{\mathrm{Hx}}_{k}$$

Equation (14) means an error between measurement value and state value. Theoretical estimate value of error covariance is yielded by deriving Equation (15):

(15) $${\hat{R}}_{k}=\frac{1}{N-1}\sum_{i=0}^{N-1}{\left[\upsilon_{i}-\bar{\upsilon }\right]}^{T}\left[\upsilon_{i}-\bar{\upsilon }\right]$$

*ῡ* is average of *v*, and it could be presented as Equation (16):

(16) $$\bar{\upsilon }=\frac{1}{N}\sum_{i=0}^{N-1}\;\upsilon_{i}$$

The discrepancy between the predicted measurement and the actual measurement *R* is called the measurement residual. A residual of zero means that the two are in complete agreement. We use the residual as a performance index of a multi-layer neural network model with the mean-square error estimator [9]:

(17) $$e_{\mathrm{Rk}}=R_{k}+{\hat{R}}_{k}$$

**Figure 4.**
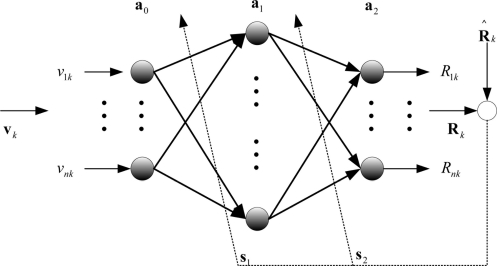
Neural network for evaluating measurement noise covariance.

A neural network can obtain the optimal noise covariance estimate *R̂_k_* by learning. The measurement noise *υ_k_*, could be used as input values of neural network. Using the measurement noise covariance *R_k_* is possible to substitute for the output of the neural network. In the proposed algorithm, the structure of the neural network is shown as Figure 4.

In the proposed algorithm, the structure of the neural network is a multi-layer neural network model. We used the error back propagation algorithm (EBA) [9]. To minimize the performance index, the EBA controls the strength of the links of each layer. The performance index selects the mean square error as the covariance error value:

(18) $$F(e)=E\left[{\left\{R_{k}-{\hat{R}}_{k}\right\}}^{T}\left\{R_{k}-{\hat{R}}_{k}\right\}\right]$$

In Equation (18), *R̂_k_* means a target value vector. The term *R_k_* is the output vector of the neural network and E is an operator of expected value. Firstly, performing EBA is disseminated an input signal to the neural network and could be presented as in Equation (19):

(19) $$a_{0}=\upsilon_{k},\;\;a_{1}=f_{1}\left(W_{1}\;a_{0}+b_{1}\right),\;\;R_{k}=a_{2}$$

In this case, *υ_k_* is an input vector of the neural network, *a*_0_ is the output vector of the input-layer in the neural network, and *a*_1_ is the output vector of a hidden-layer neuron. *W*_1_ is a weight-sum vector, and *b*_1_ is the bias vector of the hidden-layer output vector. *a*_2_ indicates the output vector of the output-layer in the neural network. *R_k_* is the output value of the neural network, and *f*_1_ is a nonlinear function of the hidden-layer neurons. The hidden-layer function is a log-sigmoid function and could be expressed as in Equation (20):

(20) $$f(n)=\frac{1}{1+e^{-n}}$$

In this case, n is pure linear input value. The original network utilized multiple layers of weight-sum units of the type *a*_1_ = *f*_1_(*W*_1_*a*_0_ + *b*_1_), where f was a sigmoid function or logistic function such as used in logistic regression. Training was done by a form of stochastic gradient descent. The use of the chain rule of differentiation in deriving the appropriate parameter updates results in an algorithm that seems to “back propagate errors”, hence the nomenclature. However it is essentially a form of gradient descent. Determining the optimal parameters in a model of this type is not trivial, and local numerical optimization methods such as gradient descent can be sensitive to initialization because of the presence of local minima of the training criterion:

(21) $$\begin{array}{cc} s_{1}=-2{\dot{F}}_{1}\left(n_{1}\right){\left(W_{2}\right)}^{T}\;s_{2}, & s_{2}=-2{\dot{F}}_{2}\left(n_{2}\right)\left({\hat{R}}_{k}-R_{k}\right) \end{array}$$

The term *s*_2_ is the sensitivity units of the output layer neuron, and the *n*_2_ is a linear output vector of the output layer neurons. The term *R̂_k_* is a target value vector, and *R_k_* is the output vector of the neural network. The term *Ḟ*_2_(*n*_2_) is a derivative of the nonlinear function of the output layer neuron. *s*_1_ is a sensitivity unit of the input layer neuron, and *Ḟ*_1_(*n*_1_) is a derivative of the input layer neuron. Lastly, weighted value and bias are updated by the steepest descent rule. It could be formulated as Equation (22):

(22) $$\begin{array}{cc} W_{m}(k+1)=W_{m}(k)-\eta s_{m}{\left(a_{m-1}\right)}^{T}, & b_{m}(k+1)=b_{m}(k)-\eta s_{m} \end{array}$$

*W_m_*(*k* + 1) is a weight-sum vector of the nest state *m*-layer neuron. *W_m_*(*k*) is the current state weight-sum vector and the *b_m_*(*k* + 1) is a bias vector about the *m* layer neuron in the following state. *b_m_*(*k*) is a bias vector of current state, and *η* is the learning rate. The learning rate is a weighted factor which determines the degree of progress.

## 4. Simulation Results

In order to evaluate the effectiveness of the proposed method, and using ther measurement system represented in Figure 1, we conducted some simulations of the Kalman filter and the improved method with each sensor data of the multi-sensing environment. The common Kalman filter can be used to reduce the noise in a simple system [10]. Therefore, we can derive the system equation for simulations and experiments from Equation (1) represented by:

(23) $$x_{k+1}=x_{k}$$

(24) $$z=x_{k}+\upsilon_{k}$$

Since the measurement system described in Figure 1(b) is a simple measurement system, there is no system noise as we can see in the literature.

### 4.1. Simulations for the Kalman Filter

**Figure 5.**
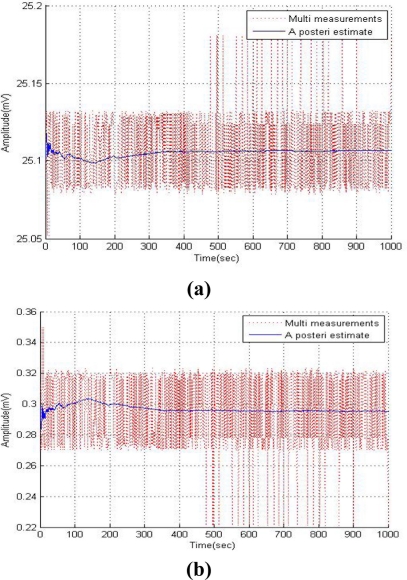

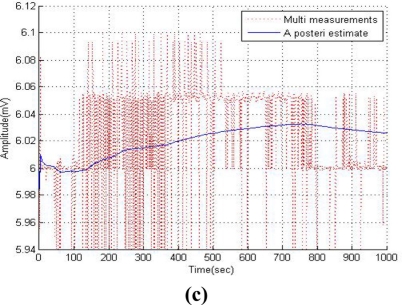
Simulation results of common Kalman filter. **(a)** Temperature sensor. **(b)** Humidity sensor. **(c)** Oxygen sensor.

To compare the results of the common Kalman method and the improved Kalman filter, in this section simulations were examined with an assumed measurement noise covariance. The Kalman filter can provide optimal solutions if the system model is correctly defined and the noise statistics for the measurement and system are completely known [7]. Figure 5 shows the simulation results with assumed measurement noise covariance 1 of the Kalman filter to evaluate the performance of the common Kalman filter. These results show that the Kalman filter method is good for reducing the measurement noise. The previous method of determining the measurement noise covariance (R) for the Kalman filter depends on the analysis of empirical data from each sensor and then modifying them, which can be described as tuning the Kalman filter. Since there are no perfect sensors, their performance varies with time and their changing environment. This causes uncertainty in the previously tuned Kalman filter method, which has a considerable impact on the performance of the Kalman filter [11].

**Figure 6.**
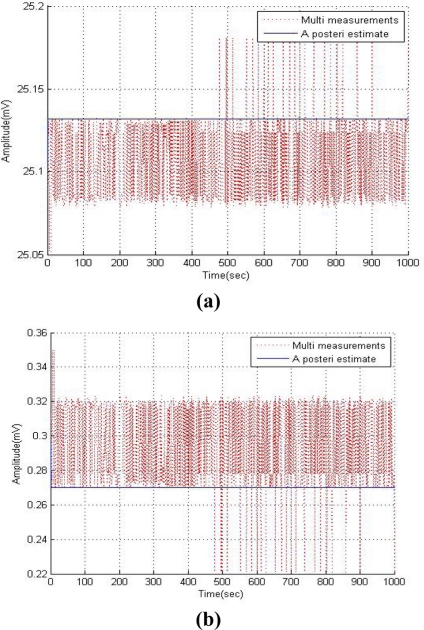

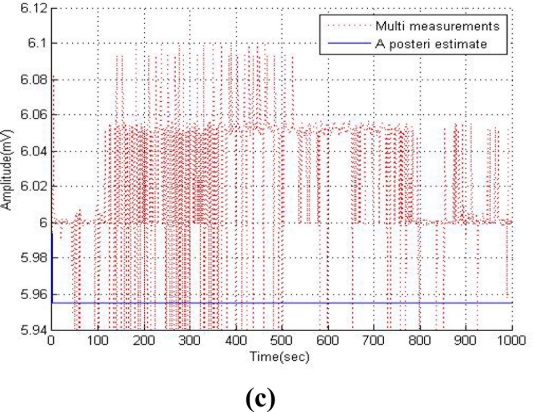
Simulation results of the Kalman filter with divergence condition. **(a)** Temperature sensor. **(b)** Humidity sensor. **(c)** Oxygen sensor.

The R value of the Kalman filter influences the weight that the filter applies between the existing process information and the latest measurements. Mismatch in any of them may result in the filter being suboptimal or even cause divergence, as shown in Figure 6 [7], which shows measured data from each sensor in a multi-sensing environment and the filtered data with an assumed measurement noise covariance of 10.

The measurement noise covariance R is a most significant factor when designing a Kalman filter. As shown in Figure 6, the Kalman filter can diverge with an erroneous selection of R.

### 4.2. Simulations for Improved Kalman Filter

In order to evaluate the performance of the proposed method, we also conducted some simulations of a Kalman filter with computed measurement noise covariance using a neural network in this section. A complex and large neural network performs better calculations, but its calculation time is long. In the simulation of the proposed method, to decrease the calculation time the structure of the neural network is composed of a simple three-layer feed forward neural network with one hidden layer which is the most widely spread architecture type. The input layer, hidden layer and output layer had 3, 5 and 3 neurons, respectively. The activation function of the hidden nodes is chosen to be a sigmoid type function and the output nodes are linear. The design factor of a neural network can change the performance of the system and can be determined through trial and error. In this simulation, the learning rate and initial bias of neural network were set at 0.01 and 1, respectively. A random value between −1 and 1 is employed as the initial weight which was computed by a trial and error method. The input vector of the neural network is determined by the three previous measurement values. Variances of 0.01625124 at the temperature sensor, 0.00166088 at the humidity sensor and 0.00165041 at the oxygen sensor were used as target values of each neural network, respectively. The measurement noise variance of each sensor was calculated from several measurement data which was varied by the sensing environment.

**Figure 7.**
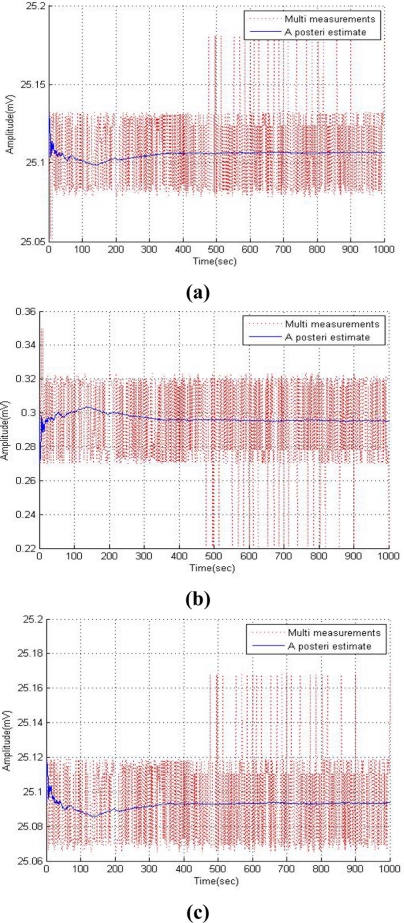
Simulation results of the improved Kalman filter. **(a)** Temperature sensor. **(b)** Humidity sensor. **(c)** Oxygen sensor.

As shown in Figure 7, the simulation results of improved Kalman filter indicate better performance than the previous method. The quality of the estimator can be assessed by the Mean Squared Error (MSE) in terms of its variation and un-biasedness. The MSE of a Kalman estimator *θ̂* referring to the estimated parameter is defined as:

(25) $$\mathrm{MSE}(\hat{\theta })=E\left[{\left(\hat{\theta }-\theta \right)}^{2}\right]$$

The MSE between measured data and *a posteri* estimated data was represented in Table 1. The improved method also represents the least MSE value. The simulation results of the Kalman method under divergence conditions which has an R value of 10 has a larger MSE value among the three cases. We can verify that the proposed method has better performance in reducing noise in a multi-sensing environment.

## 5. Experimental Results

**Figure 8.**
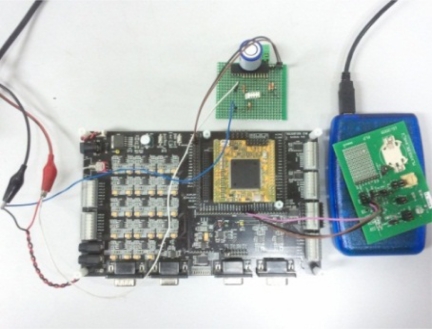
Configuration of the system for experiments.

The experiments were conducted with an EVB90129 connected with a TMS320F28X EVM with a DSPF2812 microprocessor to evaluate the proposed method in the implemented RFID sensor tag. The temperature, humidity and oxygen sensors are connected to the ADC port on the EVM board. A Serial Peripheral Interface (SPI) was used to interlock between EVB90129 and the EVM. The data from the multi-sensing environment is obtained from the organized module. In the EVM, the sensor data was filtered by the improved Kalman filter implemented on the EVM. The filtered data is transmitted to the EVB90129 and sent to a RFID reader. Figure 8 shows the measurement system for the experiments in a multi-sensing environment. The structure of the neural network was the same as in the simulation condition in those experiments.

**Figure 9.**
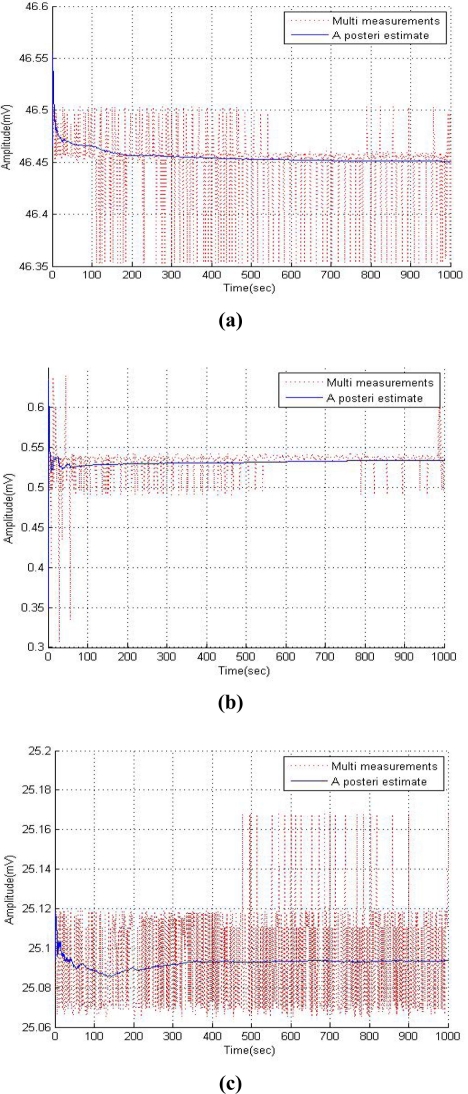
Experimental results of the improved Kalman filter. **(a)** Temperature sensor. **(b)** Humidity sensor. **(c)** Oxygen sensor.

The sensor data from the designed module was sent into the EVM. The improved Kalman filter which has simple neural network structure to evaluate the measurement noise covariance was realized in the EVM. Previous measurement data was an input of the neural network and the target value is the same as the simulation conditions. Figure 9 shows the experimental results with the system. The improved method performed was in the experimental set up. However, as shown in Table 2, the MSE between the measured data and the *a posteri* estimated data was larger than in the simulation results because of the changing actual sensing environment.

## 6. Conclusions

In order to obtain accurate sensor data in a multi-sensing environment and prevent divergence of the Kalman filter caused by disturbances of the measurement environment, we have proposed an improved Kalman filter which can estimate its measurement noise covariance using a neural network. The improved Kalman filter is realized with a neural network to estimate measurement noise covariance for preventing divergence of the Kalman filter and reduction of the measurement noise. The target value of the neural network was computed from a large number of measurement data in a multi-sensing environment, and the input is the previous measurement data. The proposed method was applied to reduce measurement noise and prevent divergence with some simulations and experiments. In the simulations of multi-sensing environments, the Kalman filter method and its divergence condition was compared to the improved Kalman filter which was proposed in this paper. From several simulation results and experimental results, the performance of the improved Kalman filter is good as excellent as those of the Kalman filter. The MSE of the improved Kalman filter were 2.4633 × 10^−4^ mV^2^, 3.7143 × 10^−4^ mV^2^ and 0.00105 mV^2^ lower than previous method with temperature sensor, humidity sensor and oxygen sensor, respectively, under the simulation conditions. The experimental results show that the MSE of the improved Kalman filter were 1.6589 × 10^−4^ mV^2^, 2.5203 × 10^−4^ mV^2^ and 0.0.00102 mV^2^ lower than those of the common Kalman filter with a temperature sensor, humidity sensor and oxygen sensor, respectively.

## Figures and Tables

**Table 1.** MSE between measured data and the *a posteri* estimated data.

**Method**	**Temperature Sensor**	**Humidity Sensor**	**Oxygen Sensor**
Common Kalman method	6.1445 × 10^−4^ (mV^2^)	6.1453 × 10^−4^ (mV^2^)	0.0012 (mV^2^)
Kalman method in divergence condition	0.0013 (mV^2^)	0.0011 (mV^2^)	0.0063 (mV^2^)
Improved Kalman method	3.6812 × 10^−4^ (mV^2^)	2.4310 × 10^−4^ (mV^2^)	0.00015 (mV^2^)

**Table 2.** MSE between measured data and the *a posteri* estimated data.

**Temperature Sensor**	**Humidity Sensor**	**Oxygen Sensor**
4.4856 × 10^−4^ (mV^2^)	3.6250 × 10^−4^ (mV^2^)	0.00018 (mV^2^)
